# Factors Predicting Information Overload During the COVID-19 Pandemic in the Digital Age: Longitudinal Study

**DOI:** 10.2196/67098

**Published:** 2025-10-30

**Authors:** Hiroko Okada, Tsuyoshi Okuhara, Takahiro Kiuchi

**Affiliations:** 1 Department of Health Communication, Schoolf of Public Health Graduate School of Medicine The University of Tokyo Tokyo Japan

**Keywords:** COVID-19, health communication, information overload, social media, infobesity, infoxication, information anxiety, data overload, SARS-CoV-2, coronavirus, respiratory, infectious, pulmonary, pandemic, digital age, information age, longitudinal study, Japan, human capacity, informational media

## Abstract

**Background:**

The human capacity to process information is limited. During the COVID-19 pandemic, people were exposed to a large amount of uncertain and complex health information. This situation made some people experience perceived information overload, which made them unable to adopt appropriate preventive behaviors.

**Objective:**

This study aimed to examine the individual characteristics, abilities, and attention to informational media that predict the perception of information overload during a pandemic.

**Methods:**

We conducted a longitudinal study with 2 time points, August 2020 and August 2021, among residents of Japan under a COVID-19 emergency declaration. The sample had the same proportions for sex, age, and prefecture as the general Japanese population. We used a web-based survey to measure sociodemographic characteristics, health literacy (HL), attention to 6 different types of information channels, and participants’ perception of information overload. Hierarchical multiple regression analysis was conducted with information overload as the objective variable.

**Results:**

A total of 784 participants responded to the survey at both time points, with a follow-up rate of 78.4% (784/1000). Hierarchical multiple regression analysis showed that younger age (β=−0.084, 95% CI −0.142 to −0.013), male sex (β=−0.163, 95% CI −0.008 to −0.003), lower HL (β=−0.084, 95% CI −0.114 to −0.011), paying less attention to television news (β=−0.118, 95% CI −0.038 to −0.001), and paying greater attention to social media (β=0.089, 95% CI 0.000-0.027) significantly predicted information overload 1 year after exposure to information during the pandemic.

**Conclusions:**

Public health communicators should aim to provide concise and understandable information in consideration of a target population that is vulnerable to information overload during a pandemic. A high level of attention to social media may increase the perception of information overload. By contrast, HL may reduce the cognitive load in information processing. Providing an environment during normal periods that allows people to develop the skills to critically interpret health information will help them to prepare for future infodemics.

## Introduction

### Background

The unexpected occurrence of the COVID-19 pandemic caused a serious and prolonged threat to human health and life. To cope with this threat, people consumed information from diverse media sources to stay informed about this emerging infectious disease [1,2]. At the 2020 Munich Security Conference, the World Health Organization (WHO) director general stated that the world was not only dealing with a pandemic, but also with an infodemic [3,4]. The COVID-19 pandemic occurred amid the ongoing proliferation of new media platforms, such as social media, where anyone can disseminate any type of information. Fake news, misinformation, and conspiracy theories spread faster than ever via these new media channels [3,5,6]. In addition, information during a pandemic is often uncertain. It has become apparent that ensuring that the public can adequately perceive information to protect their own lives and well-being during a pandemic is an enormous challenge within public health communication.

### Information Overload

The human capacity to process information is limited [7-10]. Therefore, a rapid influx of information can impose a psychological burden. Information overload can be defined as a situation where the amount of information provided within a certain time frame exceeds an individual’s capacity to process the information [11]. Information overload affects people’s decision-making accuracy, time to reach a decision, and general performance [12]. More specifically, with information overload, a person can become overly selective, ignore large amounts of information, have difficulty understanding the relevance of details with respect to the overall perspective, and require more time and effort to acquire information [12]. Similarly, when people are faced with overly complex health information, they may not recognize the information as personally relevant or valuable, and may be unable to infer what information is needed to engage in appropriate behavior [13].

### Information Overload and Health Behavior

People experiencing information overload tend to have negative attitudes toward health information [14,15]. Consequently, they are less motivated to seek out and digest such information, which ultimately reduces their health knowledge and associated skills [15,16]. In the field of health, the problem of information overload has been studied in recent years. For example, a study of cancer information seeking showed that information overload leads to the avoidance of cancer screening and a lack of appropriate preventive behavior [17]. A study of patients with newly diagnosed coronary artery disease suggested that information overload has a direct negative impact on their intention to read self-management materials [18]. Previous cross-sectional studies conducted during the COVID-19 pandemic showed that information overload is associated with vaccine hesitancy [19,20]. Information overload in the COVID-19 era has also been reported to cause negative emotions, such as anxiety and depression [21,22]. These findings show that information overload is a health communication concern as a factor that influences health behavior.

### Predictors of Information Overload: Theoretical Framework

Cognitive load theory is an important framework for understanding the causes of information overload [23]. This theory was originally introduced in the field of educational psychology and has contributed to the development of educational environments for the retention of knowledge and information [24]. As an application of learning theory, it is also useful for understanding the process by which humans acquire knowledge from news and other sources. In this theory, cognitive load is composed of 3 conceptualized factors: intrinsic, extraneous, and germane load [24]. Intrinsic cognitive load is related to the natural complexity of the information or learning materials that must be understood with respect to a specific topic. Extraneous load can be expressed as the load imposed by information from suboptimal media. When extraneous cognitive load is high, the information seeker disproportionately uses their working memory (WM) resources to deal with the provided media rather than with information seeking itself, so the attention devoted to essential information is reduced. Germane load refers to the WM resources that the information seeker expends to deal with the intrinsic cognitive load of the information. Germane load is related to individual characteristics [24].

Not all individuals experience information overload. The relationship between age and cognitive function, an individual characteristic, has been the focus of research in the fields of neuroscience and gerontology [25-27]. Related studies have shown that cognitive function declines with age in adulthood [28,29]. This suggests that the cognitive load from processing complex information in adulthood is greater with older age. However, a cross-sectional study conducted in Korea during the COVID-19 pandemic found no association between age and information overload [30]. The relationship between age and information overload in the context of health information and pandemics has not been clarified. However, evidence from the field of neuroscience shows that the cognitive load of information increases with age. It is likely that older people were more likely to perceive information overload during the pandemic, when they were exposed to a large amount of complex health information.

Our first hypothesis (hypothesis 1) is that older adults are more likely to experience information overload 1 year later in a pandemic information environment.

Previous studies have indicated that men and women have different psychological adaptations to extreme situations, such as pandemic lockdowns [31]. The WHO has emphasized the differential impact of a pandemic on men and women and the need for research on the effects of the COVID-19 pandemic from a sex-based perspective [32]. The relationship between information overload and sex has been reported in contexts of exposure to news. These studies have shown that women tend to experience greater information overload than men owing to news exposure [4,33,34]. It has been found that men and women use different cognitive resources with respect to news story structures, with women having slower reaction times to news stories [35]. The sex differences in cognitive load are also supported by research reporting functional magnetic resonance imaging data [26]. When men and women were given the same verbal and visuospatial WM tasks, women experienced greater task demands and higher cognitive load than men. However, as mentioned, the relationship between sex and information overload in the context of health information and pandemics remains unclear. Studies conducted in the context of exposure to early COVID-19 and cancer information reported no significant differences in the perception of information overload between men and women [36]. These studies also had limitations, such as having a cross-sectional design or having limited participant demographics. In the context of the COVID-19 pandemic, news continues to be disseminated daily. Evidence regarding the processing of WM tasks and news suggests that women may be more likely to recognize information overload in the information environment within a pandemic context.

Our first hypothesis (hypothesis 2) is that women are more likely to experience information overload 1 year later in a pandemic information environment.

Health information frequently contains complex medical wording [37,38]. This can especially affect the processing of information, especially for people with reading and writing difficulties [39]. Furthermore, the information released during a pandemic is fraught with uncertainty and is continually revised as new findings emerge. People must engage in repeated information processing each time information is updated. The intrinsic cognitive load caused by this complexity of information is affected by the learner’s previous psychological state and knowledge [23]. Therefore, it has been reported that having relevant previous specialist knowledge reduces the learner’s intrinsic cognitive load [40]. The degree to which an individual is able to find, understand, and use information and services that are useful for making decisions and taking action regarding their health is referred to as health literacy (HL). There are 3 aspects to HL. Functional HL refers to basic reading and writing skills, communicative HL refers to the ability to extract information from various forms of communication and apply it to changing situations, and critical HL refers to the ability to critically evaluate and use information to exert greater influence [41]. HL has been noted to be related to the experience of information overload as well as health status [41]. For example, previous studies conducted in the context of information seeking regarding cancer and weight management have reported that lower HL correlates with a higher perception of health information overload [42-44]. A cross-sectional study conducted in the United States during the COVID-19 pandemic also reported that individuals with lower HL were more likely to perceive information overload [45]. It is rational to consider that longitudinal analyses would similarly find that high levels of HL are likely to prevent information overload.

Our first hypothesis (hypothesis 3) is that people with higher HL are less likely to experience information overload 1 year later in a pandemic information environment.

When faced with an unknown infectious disease, people pay attention to information provided through various types of media to fill gaps in their knowledge [1,2]. It has been reported that processing information presented by diverse media sources is cognitively difficult [46]. There have been various reports on the impact of social media on users’ cognitive load. For example, there are reports on intrinsic cognitive load factors, such as fear of missing out (FOMO), privacy concerns, anxiety, and depression [47]. There have also been reports of extrinsic cognitive load factors, such as parental influence, cyberbullying, complexity, technology-related factors, and social overload [48]. In a study examining news attention and information overload, television viewing lowered the perception of information overload, whereas the use of a PC or Facebook increased perceptions of information overload [33]. During the COVID-19 pandemic, social media has been used daily as a platform for information exchange [49]. Social media users are constantly searching, receiving, identifying, reading, and processing a continuous flow of information. Users have been reported to experience a drain on cognitive energy, feeling overwhelmed and fatigued by the cognitive demands of social media [48,50]. Several cross-sectional studies have reported that people who follow COVID-19-related information on social media are more likely to experience information overload [51,52]. It is rational to assume that a longitudinal analysis would find that attention to social media leads to a greater perception of information overload than exposure to old media.

Our first hypothesis (hypothesis 4) is that greater exposure to social media information increases the experience of information overload 1 year later in a pandemic information environment.

The information flow during a pandemic differs in quantity and quality compared with that during a normal search for information on diseases, such as cancer. There is a need to identify the characteristics of individuals and information channels that predict information overload during a pandemic. Combined with skills that can reduce cognitive load owing to complex information, this information would provide important resources for the design of public health communication strategies to increase appropriate preventive behaviors. However, to the best of our knowledge, no previous studies have longitudinally examined the predictors of information overload during the COVID-19 pandemic.

### This Study

The purpose of this study was to longitudinally examine predictors of information overload during the COVID-19 pandemic in terms of individual characteristics, health information–handling skills, and exposure to information as an available resource for information processing. Identifying the factors that influence information overload is essential to the theoretical understanding of these associations and to improving the efficiency of public health communication.

## Methods

### Study Design

We conducted a longitudinal study in Japan using a web-based survey administered at 2 time points: in August 2020, soon after the WHO’s declaration of the COVID-19 pandemic, and 1 year later, in August 2021. The state of emergency declared by the Japanese government was in effect at both times.

### Data Collection

A sample was drawn from a panel of 2.2 million Japanese residents using the platform of the research firm Rakuten Insight, Inc. This study was conducted as part of a multipurpose longitudinal survey to examine multiple research questions during the pandemic. We used nonprobabilistic quota sampling for age, gender, and place of residence to match the Japanese population distribution. The sample had a similar distribution for sex, age, and prefecture as the general Japanese population. People eligible to participate in the study were those (1) who lived in areas where a state of emergency had been declared at our study time points, (2) who were not infected with COVID-19 and had no family members who were infected, and (3) who were aged 18 years or older.

We conducted the first survey (T1) on August 15 and 16, 2020, and the second survey (T2) on August 15 to 17, 2021. The 1820 members of the survey panel who met the inclusion criteria were invited via email to respond to the screening questions. As part of the screening process, participants were required to read a web page that outlined our research. After agreeing to participate, participants were given a URL to access the online questionnaire, and then they completed the questionnaire manually. This survey was a closed survey that required participants to enter their ID and password to complete. The survey form developed in this study was sent to 10 participants who had been recruited using the convenience sampling method, and we checked for any items that were difficult to answer or inappropriate before the main survey. Using adaptive questioning, we minimized the number of questions displayed to individuals. All questions displayed were mandatory, and the next question was displayed after the participant had completed the previous question. There was a page where participants could review their answers after completing all the questions, and they could make corrections before submitting their answers.

The T1 closed when 1000 participants from 8 prefectures (Chiba, Hokkaido, Hyogo, Kanagawa, Kyoto, Osaka, Saitama, and Tokyo) completed the questionnaire. The T2 was conducted among the participants recruited in T1. The areas surveyed were under a state of emergency during both survey periods. An analysis was conducted among the 784 participants who completed both surveys. The follow-up rate was 78.4% (784/1000).

### Measurements

#### Independent Variables

##### Sociodemographic Data

Data on age (continuous), sex (0=male, 1=female), level of education (categorical), household income (categorical), history of infection (binary), and cohabiting family (binary) were collected during T1.

##### Health Literacy

HL was measured using the 5-item version of the Communicative Critical Health Literacy instrument developed by Ishikawa et al [53]. This instrument uses a 5-point Likert scale ranging from 1 (completely disagree) to 5 (strongly agree) and was applied in T1. The score was the average of 5 items (continuous variables), with higher scores indicating higher HL. The SD in this study was 0.6. The Cronbach α in this study was 0.88.

##### Attention Paid to Information Channels About the COVID-19 Pandemic

The T1 assessed the frequency of exposure to 7 types of media information channels: newspapers, television news, television tabloid shows, news websites, official websites of the government and medical professional organizations, video-sharing sites, and social media. For each channel, respondents were asked a single question about the extent to which they had paid attention to COVID-19–related information on that media channel over the past week (eg, “How much attention did you pay to information about COVID-19 in newspapers?”). Each item was rated on a 10-point scale that ranged from 1 (paid no attention at all) to 10 (paid a great deal of attention), which was adapted from a previous study [53]. The score was a continuous variable ranging from 1 to 10, with higher scores indicating greater attention to that information channel.

##### COVID-19 Information Overload

Information overload was assessed using the Corona Information Overload Scale developed by Sarkhel et al [54]. This scale is an adaptation of the Cancer Information Overload scale, modified for the COVID-19 setting [54]. Example items include statements, such as “There are so many different recommendations for the prevention of COVID-19 that it is difficult to know which ones I should follow.” The scale comprises 8 items, with each item rated on a 4-point Likert scale ranging from 1 (do not at all agree) to 4 (very much agree). The score is the average of 8 items (continuous variables), with higher scores indicating a greater perception of information overload. Information overload was assessed at T2. The SD in this study was 0.4. The Cronbach α in this study was 0.89.

#### Ethical Considerations

A protocol for this study was approved by the ethics review committee of the Graduate School of Medicine, University of Tokyo, based on guidelines for clinical trials involving human subjects (11270). Participants were provided with an explanation of the study and completed a web-based consent form. All participants were given the right to withdraw from the study at any time during the study period. Data were provided to researchers by the research company in a linked but anonymized form. Participants in the online survey were offered points that could be used as 1 yen (US $0.009) per point on the Rakuten e-commerce site as an incentive.

#### Statistical Analysis

Descriptive statistics were calculated for each sociodemographic variable. We then performed single linear regression analysis as a crude analysis, with the objective variables being information overload, sociodemographic characteristics, HL, and attention to media information.

Hierarchical multiple linear regression analysis was performed as an autoregressive model. In model 1, we included information overload (T2) as the outcome variable and sociodemographic characteristics, including age (continuous; sex, 0=male, 1=female), level of education (categorical), household income (categorical), history of infection (binary), and cohabiting family (binary), as independent variables. In addition to the variables in model 1, we included HL (T1) as an independent variable in model 2. In addition to the variables in model 2, we included various information channels (T1: news, television tabloid shows, newspapers, news websites, nonnews websites, and social media) as independent variables in model 3. All variables entered as independent variables for all models were forced into the model based on previous studies or theory as variables associated with information overload.

No missing values were observed because the online survey was designed such that respondents could not proceed to the next question if values were missing. Participants who only responded to T1 were not included in our analysis. All tests were 2-sided, and the significance level was set at 5%. We used IBM SPSS (version 25; IBM Corp) for the analysis.

## Results

Participants’ characteristics are presented in Table 1. The breakdown of characteristics by sex, age, and prefecture of residence was similar to that of the general population in Japan, as stated in the Methods section. The minimum age was 21 years and the maximum age was 76 years. The proportions of participants with an education level lower than a university degree and with a university degree or higher were similar. Approximately half of the participants were full-time employees. The demographic characteristics of participants who were included in the analysis did not differ statistically from the characteristics of those who were excluded owing to not having completed T2.

**Table 1:** The characteristics of the study participants (N=784).

Participant characteristics			Values
Age (y), mean (SD)			47.14 (12.8)
**Sex,** **n (%)**			
	Male	396 (50.5)	
	Female	388 (49.5)	
**Education,** **n (%)**			
	Junior high school	12 (1.5)	
	High school	156 (19.9)	
	Vocational school or junior college	175 (22.3)	
	University	379 (48.3)	
	Graduate school	62 (7.9)	
**Household income,** **n (%)**			
	<JP ¥2,000,000 (<US $15,000)	64 (8.2)	
	JP ¥2,000,000-¥6,000,000 (US $15,000-$43,500)	320 (49)	
	>JP ¥6,000,000 (>US $43,500)	334 (42.6)	
	Unclear	66 (8.4)	
Previous infection, n (%)			21 (2.7)
Cohabiting with family, n (%)			613 (78.2)
Health literacy^a^, mean (SD)			3.822 (0.6)

^a^Health literacy was measured by the Communicative Critical Health Literacy.

Table 2 shows the results of the single regression analysis of sociodemographic characteristics, HL, and attention to media information channels in terms of COVID-19 information overload. Significant simple associations with information overload were found for age, sex, education, HL, television news, television tabloid shows, newspapers, and social media.

**Table 2:** Univariate associations between information overload and explanatory variables (N=784).

	B	β (SE; 95% CI)	*P* value^a^
Age (y)	−0.006	−0.154 (0.001; −0.008 to −0.003)	<.001
Sex	−0.061	−0.067 (0.033; −0.125 to 0.003)	.06
Education	−0.009	−0.018 (0.017; −0.043 to 0.025)	.61
Household income	−0.052	−0.070 (0.027; −0.104 to 0.000)	.051
Cohabiting with family	−0.057	−0.051 (0.040; −0.134 to 0.021)	.15
Previous infection	−0.286	−0.059 (0.174; −0.627 to 0.054)	.10
Health literacy^b^	−0.082	−0.111 (0.026; −0.133 to −0.030)	.002
Television news	−0.030	−0.186 (0.006; −0.042 to −0.019)	<.001
Television tabloid show	−0.022	−0.137 (0.006; −0.032 to −0.011)	<.001
Newspaper	−0.019	−0.138 (0.005; −0.029 to −0.009)	<.001
News website	−0.017	−0.093 (0.006; −0.030 to −0.004)	.009
Nonnews website	−0.010	−0.048 (0.007; −0.025 to 0.005)	.18
Social media	0.011	0.068 (0.006; 0.000 to 0.021)	.06

^a^The estimates were calculated by simple regression analysis.

^b^Health literacy was measured by the Communicative Critical Health Literacy instrument.

Table 3 shows the results of hierarchical multiple regression analysis. The results of model 1 showed that younger age (β=−0.084, 95% CI −0.142 to −0.013) was significantly more highly correlated with information overload 1 year later. For sex, information overload was significantly higher for men than for women (β=−0.163, 95% CI −0.008 to −0.003). Lower household income was associated with significantly higher information overload (β=−0.083, 95% CI −0.115 to −0.009). The model 2 results showed that the lower the HL, the higher the information overload (β=−0.084, 95% CI −0.114 to −0.011), which was significant. As the results of model 3 show, information overload was significantly higher for those who paid less attention to television news (β=−0.118, 95% CI −0.038 to −0.001) and for those who paid greater attention to social media (β=0.089, 95% CI 0.000-0.027).

**Table 3:** Multivariate associations between information overload and sociodemographic characteristics, health literacy, and COVID-19 information attention (N=784)^a^.

	Model 1				Model 2				Model 3		
	B	β (SE; 95% CI)	*P* value^b^	B		β (SE; 95% CI)	*P* value^b^	B		β (SE; 95% CI)	*P* value^b^
Age (y)	−0.006	−0.158 (0.001; −0.008 to −0.003)	<.001	−0.005		−0.145 (0.001; −0.008 to −0.003)	<.001	−0.003		−0.078 (0.001; −0.006 to 0.000)	.054
Sex	0.076	0.083 (0.033; 0.011 to 0.140)	.022	0.074		0.081 (0.033; 0.009 to 0.138)	.025	0.072		0.079 (0.033; 0.008 to 0.136)	.03
Education	−0.016	−0.033 (0.018; −0.051 to 0.019)	.373	−0.012		−0.026 (0.018; −0.048 to 0.023)	.488	−0.004		−0.008 (0.018; −0.039 to 0.031)	.82
Household income	−0.059	−0.080 (0.029; −0.116 to −0.003)	.040	−0.056		−0.076 (0.029; −0.113 to 0.000)	.051	−0.051		−0.069 (0.029; −0.107 to 0.005)	.08
Cohabiting with family	−0.012	−0.011 (0.042; −0.093 to 0.070)	.778	−0.015		−0.014 (0.042; −0.097 to 0.066)	.714	0.010		0.009 (0.042; −0.072 to 0.092)	.80
Previous infection	−0.221	−0.046 (0.172; −0.559 to 0.117)	.199	−0.209		−0.043 (0.172; −0.546 to 0.128)	.224	−0.210		−0.043 (0.171; 0.546 to 0.127)	.22
Health literacy^c^	—^d^	—	—	−0.062		−0.083 (0.026; −0.113 to −0.01)	.019	−0.061		−0.082 (0.026; −0.112 to 0.009)	.02
Television news	—	—	—	—		—	—	−0.019		−0.117 (0.010; −0.038 to 0.000)	.045
Television tabloid show	—	—	—	—		—	—	0.002		0.011 (0.009; −0.015 to 0.018)	.84
Newspaper	—	—	—	—		—	—	−0.006		−0.042 (0.006; −0.017 to 0.006)	.32
News website	—	—	—	—		—	—	−0.007		−0.036 (0.008; −0.022 to 0.008)	.39
Nonnews website	—	—	—	—		—	—	−0.012		−0.056 (0.009; −0.030 to 0.007)	.22
Social media	—	—	—	—		—	—	0.014		0.089 (0.007; 0.000 to 0.027)	.048

^a^For model 1, R2 was 0.039, ΔR2 was 0.039, *F*_6,777_=5.292, and *P* value was <.001. For model 2, R2 was 0.046, ΔR2 was 0.007, *F*_1,776_=5.490, and *P* value was *.*02. For model 3, R2 was 0.072, ΔR2 was 0.026, *F*_1,770_=3.532, and *P* value was .002.

^b^The estimates were calculated by hierarchical multiple regression analysis.

^c^Health literacy was measured by the Communicative Critical Health Literacy instrument.

^d^Not applicable.

## Discussion

### Overview

In this study, we examined predictors of information overload during the COVID-19 state of emergency declaration in Japan. The results suggest that predictors of information overload include individual characteristics, skills for handling health information, and the information channels to which individuals are exposed. These results are important findings for developing public health communication strategies that take into account people who are more likely to perceive information overload during a pandemic.

### Individual Characteristics That Predict Information Overload

The results of this study did not support our hypothesis that older people are more likely to experience information overload during a pandemic. There are 2 possible reasons why younger people were more likely to experience information overload during the prolonged COVID-19 pandemic conditions. First, young people had fewer health problems before the pandemic than older people. As a result, younger people may have had less experience searching for and processing health information themselves. Previous studies have reported that people with less experience in information processing are more likely to perceive information overload [12,14]. When appropriate resources are lacking, it becomes difficult to integrate new information with an individual’s existing knowledge; therefore, the individual does not know how to react to new information [14]. Second, new media channels, such as social media, have developed rapidly in recent years. Younger people are likely to be exposed to older types of media but also use multiple new media channels [55]. During the COVID-19 pandemic, people were exposed to a greater volume of information from a variety of sources, media, and platforms than they were during previous pandemics [52]. This diversity and complexity of platforms can lead to exposure to irrelevant information and the need to allocate extra WM [40,48,56]. This may have resulted in a greater cognitive burden on younger people. The results of this study suggest that, in the special information environment of a pandemic, young people may experience greater cognitive load than older people.

Contrary to our hypothesis, men were more likely to perceive information overload than women in this study. Although previous studies have demonstrated sex differences in behaviors related to the COVID-19 pandemic, the relationship between information overload and sex has been unclear [31,36]. During a prolonged pandemic, people are affected psychologically and behaviorally by various external triggers. Similar to information overload, one of these triggers is pandemic fatigue, which is more often perceived by men [57]. Pandemic fatigue has been described by the WHO as a loss of motivation owing to the chronicity of a pandemic situation and reportedly comprises 2 concepts: neglect of preventive behaviors and boredom with overexposure to information [58]. Fatigue from repeated exposure to similar information is reportedly associated with information overload [59]. It is possible that men are more vulnerable than women to fatigue owing to repeated exposure to similar information.

In future pandemics, more diverse information may be disseminated than during the COVID-19 pandemic, and more people may perceive information overload. As the results of this and previous studies have shown, younger people and men may not be able to acquire appropriate and correct knowledge owing to information overload and may have difficulty adopting appropriate preventive behaviors. It is necessary to consider information provision strategies that are appropriate for these populations.

### Effect of Health Information–Handling Skills With Information Overload

The results of this study support the hypothesis that people with higher levels of HL are less likely to experience information overload. This finding is consistent with the results of 2 cross-sectional studies conducted in the United States during the COVID-19 pandemic [45,59]. Those results suggest that previous skills in handling health information may reduce the intrinsic cognitive load of complex information in a special information environment, such as a pandemic [40]. The HL scale used in our study measured 2 aspects of HL: communicative literacy, which is the ability to extract information and derive meaning, and critical literacy, which is the ability to critically analyze information. It has been reported that critical thinking is an important skill in mitigating information overload [60]. Critical thinking is closely related to information mastery competencies, such as problem-solving and self-management [61].

As a skill that individuals can develop in advance, HL is an important resource to help combat information overload during a pandemic. HL can be improved through intervention [62]. Therefore, education in developing the skills needed to appropriately process health information should be promoted. Although not addressed in this study, functional literacy is also an important aspect of HL. The variance in functional literacy among Japanese people is small, and it is not suitable for predicting health-related behavior and outcomes. However, additional findings on this aspect will lead to the development of information provision strategies for people with limited functional HL [63].

### Information Channels That Predict Information Overload

Regarding the association between the degree of attention to each information channel and information overload, our study results support our hypothesis that greater attention to social media information is a predictor of information overload. In addition, we found that survey respondents who were attentive to television news had a significantly lower perception of information overload. This result can be explained by the characteristics of the information provided by both types of media, as follows. First, the characteristics of the media content differ between these types of media. Television news involves mainly objective facts that have been scrutinized by the news organization, whereas social media information involves mainly subjective perspectives and interpretations by individuals. The 2018 Edelman Trust Barometer Special Report concluded that only 41% of people trust social media [64]. When obtaining information from another individual on social media, there is a cognitive load involved in determining whether that information is worth believing [55]. Concerns about the quality of information may contribute to increasing the cognitive load in information processing [43,65]. A cross-sectional study conducted in the United States during the COVID-19 pandemic reported that individuals receiving information through 2 channels, social media and interpersonal communication, were less likely to perceive information overload than those receiving information through social media alone [65]. Social media information may reduce the cognitive load when provided through interpersonal communication. Second, the originator of information differs among these media sources. Television news is transmitted from a news broadcasting agency by professional news readers, whereas social media information can be transmitted by anyone, including laypersons, and is often put forth by people who are closer and more connected to the individual receiving the information. One cause of the intrinsic cognitive load with social media is FOMO, which can occur when people exchange information among connected individuals [66]. FOMO is defined as a person’s experience of pervasive anxiety that others may be having rewarding experiences that they themselves are not experiencing, and is characterized by a desire to continuously connect with what others are doing [67]. This makes it difficult for some individuals to reduce or stop using social media for fear of missing important information and activity on social media [68,69]. From the perspective of cognitive load, personal informational media to which the user has an emotional attachment (eg, social media) may involve a high cognitive load, whereas informational media that provides objective information with no emotional attachment (eg, television news) may produce a lower cognitive load.

On the basis of the results of this research and the findings of previous studies, we propose several approaches to reducing information overload during a pandemic from the perspectives of intrinsic, extrinsic, and germane cognitive load. First, health information about a pandemic is uncertain and changes over time, which means it has a high intrinsic load. One factor that reduces intrinsic load is previous specialist knowledge and skills. Encouraging people to develop skills, such as HL, information literacy for reading information critically, and slow reading under normal circumstances, will help to reduce the cognitive load caused by the complexity of information during a pandemic [60,70]. Approaches to reducing extrinsic load using technological methods have been proposed. These include methods that improve conciseness, consistency, and understandability of information content, as well as increasing findability, a technique that makes it easier for users to find content based on their needs [60,71]. Finally, for people who have characteristics that make them more susceptible to cognitive overload from complex health information during a pandemic, it may be possible to provide information that is less likely to cause overload by segmentation according to sex and age. These approaches can be adopted as part of an information delivery strategy by public health communicators to mitigate information overload during a pandemic.

### Limitations

Several limitations should be considered when interpreting the results of this study. First, because this was an observational study, a causal relationship between individual characteristics or attention to media information and information overload cannot be established from these results. Therefore, we relied on previous studies and existing theories in our discussion. However, given the considerable difficulty in conducting a randomized controlled trial during a public health crisis, the results of this study represent important findings. Second, study participants were registered members of a survey panel recruited by an internet company, and their educational background tended to be higher than that of the general Japanese public. We adjusted for the influence of educational background using multiple regression analysis; however, there is a possibility that there are still unadjusted factors related to self-selection. In addition, considering the R2 scores of each regression model, there may be unknown variables that can better explain information overload. Finally, single-item, unvalidated scales were used in this study to assess the degree of attention to media information. We adopted items that have been commonly used in previous studies to enable comparisons with results from other infectious disease pandemics and findings from other countries. In the future, we anticipate the development of scales that will measure these factors.

### Conclusions

During the COVID-19 pandemic, people have been exposed to a great deal of information through multiple media channels, constituting an infodemic. The results of this study have longitudinal implications in terms of the characteristics of individuals and types of informational media that can predict information overload in a specific informational environment. Public health communicators should consider younger people, men, and people with low HL when developing public health communication strategies during a pandemic. High levels of social media attention may increase the perception of information overload. Therefore, public health communicators can incorporate technical approaches to prevent information overload during a pandemic, such as improving the findability of appropriate information and preparing concise, understandable content. Moreover, providing opportunities for educating the public about critical reading of health information during normal periods will help individuals to prepare for future infodemics during which anyone can disseminate information.

## Abbreviations

FOMO: fear of missing out
HL: health literacy
T1: first survey
T2: second survey
WHO: World Health Organization
WM: working memory

## Data Availability

The datasets generated or analyzed during this study are available from the corresponding author on reasonable request.
